# Overstimulation of NMDA Receptors Impairs Early Brain Development *in vivo*

**DOI:** 10.1371/journal.pone.0036853

**Published:** 2012-05-11

**Authors:** Tomomi Aida, Yoshimasa Ito, Yuko K. Takahashi, Kohichi Tanaka

**Affiliations:** 1 Laboratory of Molecular Neuroscience, School of Biomedical Science and Medical Research Institute, Tokyo Medical and Dental University, Tokyo Japan; 2 CREST, Japan Science and Technology Agency, Kawaguchi, Japan; Universitat Pompeu Fabra, Spain

## Abstract

**Background:**

Brains of patients with schizophrenia show both neurodevelopmental and functional deficits that suggest aberrant glutamate neurotransmission. Evidence from both genetic and pharmacological studies suggests that glutamatergic dysfunction, particularly with involvement of NMDARs, plays a critical role in the pathophysiology of schizophrenia. However, how prenatal disturbance of NMDARs leads to schizophrenia-associated developmental defects is largely unknown.

**Methodology/Principal Findings:**

Glutamate transporter GLAST/GLT1 double-knockout (DKO) mice carrying the NMDA receptor 1 subunit (NR1)-null mutation were generated. Bouin-fixed and paraffin-embedded embryonic day 16.5 coronal brain sections were stained with hematoxylin, anti-microtubule-associated protein 2 (MAP2), and anti-L1 antibodies to visualize cortical, hippocampal, and olfactory bulb laminar structure, subplate neurons, and axonal projections. NR1 deletion in DKO mice almost completely rescued multiple brain defects including cortical, hippocampal, and olfactory bulb disorganization and defective corticothalamic and thalamocortical axonal projections.

**Conclusions/Significance:**

Excess glutamatergic signaling in the prenatal stage compromises early brain development via overstimulation of NMDARs.

## Introduction

While multiple models have been put forth regarding the pathophysiology of schizophrenia, the vast majority of evidence suggests that schizophrenia stems from neurodevelopmental deficits, resulting in disturbance of glutamatergic neurotransmission and especially NMDA receptor-mediated signaling many years later [1], [2]. NMDAR dysfunction models are based upon the observation that psychotomimetic agents such as ketamine and phencyclidine (PCP) induce symptoms of schizophrenia in healthy subjects and provoke relapse in schizophrenics by blocking neurotransmission at NMDA receptors [3]–[6]. In rodents, NMDAR antagonists induce schizophrenia-related behavioral abnormalities [6]–[8]. While these psychotomimetic effects of NMDAR antagonists have fostered the notion of a hypoglutamatergic state in schizophrenia, recent data suggest that these effects are linked to a loss of NMDAR-mediated GABAergic inhibition, leading to excessive glutamate release and neuronal hyperexcitability in the prefrontal cortex (PFC) [2]. In support of this model is the recent demonstration of the antipsychotic efficacy of group II metabotropic glutamate 2/3 (mGlu2/3) agonists, which decrease glutamate release and normalize NMDAR antagonist-induced increases in PFC glutamate [9]. These developments suggest that elevation in the cellular balance of excitation and inhibition within the PFC may be involved in the pathophysiology of schizophrenia [10].

According to the neurodevelopmental model, the etiology of schizophrenia may involve pathologic processes caused by both genetic and environmental factors that begin before the brain approaches its adult anatomical state in adolescence. Multiple lines of evidence from brain pathology, genetics, environmental factors, and gene-environment interactions support this neurodevelopmental model [1]. Numerous reports document the presence of various neuropathological findings in schizophrenia patients, including ventricular enlargement, reduced white and gray matter diffusion anisotropy, and abnormal laminar organization [1], [11]–[13]. At the perinatal stage, a major risk for schizophrenia is birth complications, especially perinatal hypoxia [1]. Since hypoxia impairs energy-dependent glutamate transport, allowing extracellular glutamate to reach excitotoxic levels [14], it is possible that increased NMDAR activity caused by excessive glutamate plays a role in the neurodevelopmental deficits of schizophrenia.

We recently generated mutant mice in which glutamate receptors are overstimulated by knocking out glutamate transporters GLAST and GLT1, which are essential for maintaining low extracellular glutamate levels [15]. GLAST/GLT1 double-knockout (DKO) mice demonstrate multiple brain defects that are similar to schizophrenia-associated developmental defects, including enlarged lateral ventricles; disorganization of neocortex, hippocampus, and olfactory bulb due to impaired neuronal migration; and defective corticothalamic and thalamocortical axonal projections [15]. All glutamate receptor subunit classes, including NMDA, AMPA, kainite, and metabotropic receptors, are widely expressed throughout the embryonic brain [16]–[19]. To confirm the involvement of excess NMDAR signaling in these developmental defects, we generated DKO mice carrying the NMDA receptor 1 subunit (NR1)-null mutation (triple knockout, TKO) [20]. NR1 deletion in DKO mice almost completely rescued multiple brain defects including cortical, hippocampal, and olfactory bulb disorganization and defective corticothalamic and thalamocortical axonal projections.

**Figure 1:**
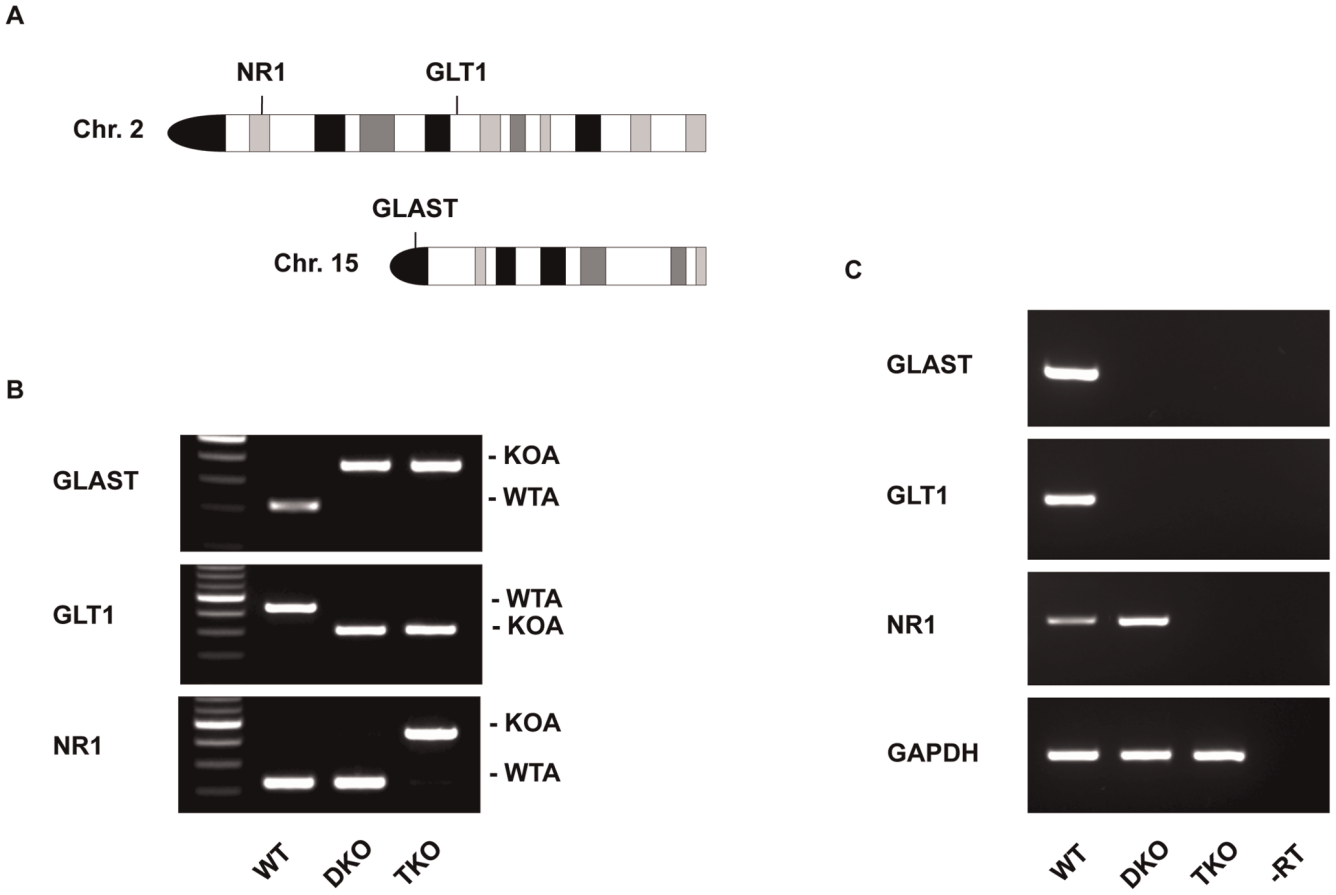
NMDA receptor 1 subunit (NR1) is deleted in the GLAST/GLT1 knock-out (DKO) mice. (**A**) The mouse GLAST gene is located on chromosome 15, whereas the mouse GLT1 and NR1 genes are both on chromosome 2, with an allelic distance of 37 cM. The idiogram is obtained from David Adler’s Idiogram Album (http://www.pathology.washington.edu/research/cytopages/idiograms/mouse/). (**B**) Mice were genotyped by genomic polymerase chain reaction (PCR) analysis. WT, wild type; DKO, GLAST/GLT1 double knockout; TKO, GLAST/GLT1/NR1 triple knockout; KOA, knockout allele; WTA, wild-type allele. (**C**) Reverse transcription-PCR (RT-PCR) analysis confirmed the absence of GLAST, GLT1, and NR1 in TKO brains. GAPDH fragment was used as loading control. –RT indicates gene fragment amplification without reverse transcriptase as a negative control.

**Figure 2:**
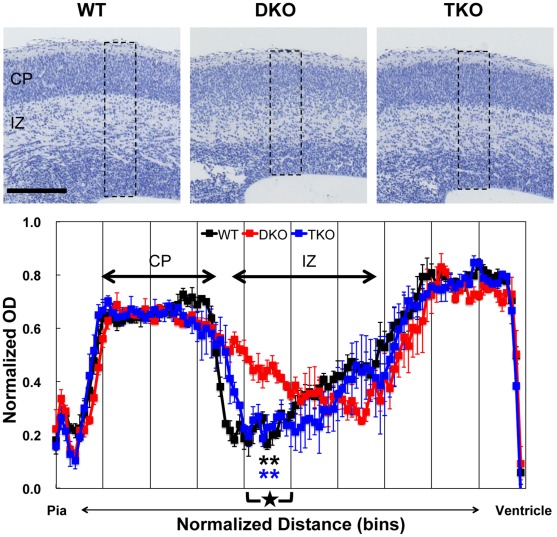
Rescue of disturbed laminar organization of cerebral cortex of DKO by deletion of NR1. (**Upper panels**) NR1 deletion in DKO rescued disturbed laminar organization of cerebral cortex. Paraffin-embedded E16.5 coronal brain sections were stained with hematoxylin. Scale bar: 200 µm. (**Lower panel**) Quantitative analysis of laminar organization of cerebral cortex. To quantify the optical density (OD), densitometry scans of the boxed regions (shown in upper panels, 100 µm width) were performed. The pia-ventricular extent was normalized by dividing it into 100 bins (x-axis). Average values of normalized OD in each bin (y-axis) were plotted along the pia-ventricular axis. Three independent embryos for each genotype were used (black: WT, red: DKO, blue: TKO). Data are mean ± s.e.m. Statistical significance was calculated every 10 bins (segment, x-axis scale marks). **P<0.01 compared to DKO (black: WT, blue: TKO) in segment 5 (star) by one-way ANOVA followed by Scheffé’s post-hoc analysis. CP: cortical plate, IZ: intermediate zone.

## Results

**Figure 3:**
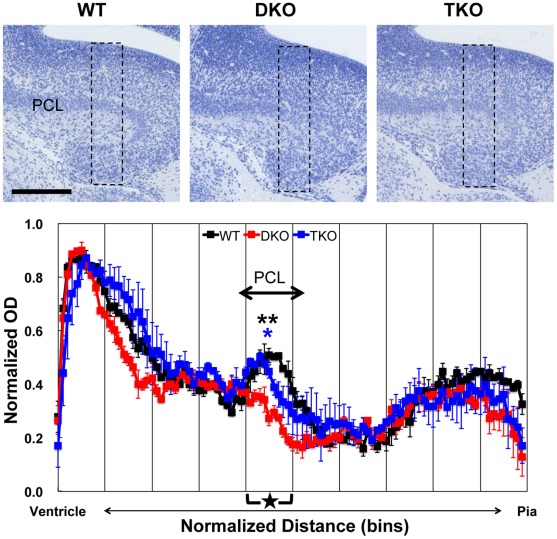
Rescue of disturbed pyramidal cell layer organization of hippocampus of DKO by deletion of NR1. (**Upper panels**) NR1 deletion in DKO rescued disturbed pyramidal cell lyaer organization of hippocampus. Paraffin-embedded E16.5 coronal brain sections were stained with hematoxylin. Scale bar: 200 µm. (**Lower panel**) Quantitative analysis of laminar organization of hippocampus. To quantify OD, densitometry scans of the boxed regions (shown in upper panels, 100 µm width) were performed. The pia-ventricular extent was normalized by dividing it into 100 bins (x-axis). Average values of normalized OD in each bin (y-axis) were plotted along the pia-ventricular axis. Three independent embryos for each genotype were used (black: WT, red: DKO, blue: TKO). Data are mean ± s.e.m. Statistical significance was calculated every 10 bins (segment, x-axis scale marks). **P<0.01 between WT and DKO and *P<0.01 between TKO and DKO in segment 5 (star) by one-way ANOVA followed by Scheffé’s post-hoc analysis. PCL: pyramidal cell layer.

**Figure 4:**
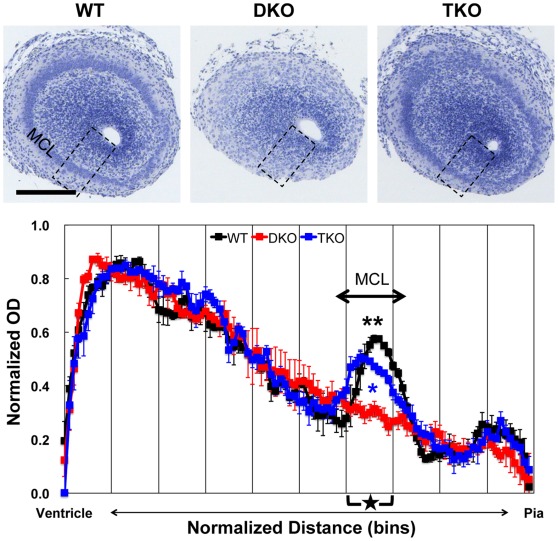
Rescue of disturbed mitral cell layer organization of olfactory bulb of DKO by deletion of NR1. (**Upper panels**) NR1 deletion in DKO rescued disturbed mitral cell lyaer organization of olfactory bulb. Paraffin-embedded E16.5 coronal brain sections were stained with hematoxylin. Scale bar: 200 µm. (**Lower panel**) Quantitative analysis of laminar organization of olfactory bulb. To quantify OD, densitometry scans of the boxed regions (shown in upper panels, 100 µm width) were performed. The pia-ventricular extent was normalized by dividing it into 100 bins (x-axis). Average values of normalized OD in each bin (y-axis) were plotted along the pia-ventricular axis. Three independent embryos for each genotype were used (black: WT, red: DKO, blue: TKO). Data are mean ± s.e.m. Statistical significance was calculated every 10 bins (segment, x-axis scale marks). **P<0.01 between WT and DKO and *P<0.05 between TKO and DKO in segment 5 (star) by one-way ANOVA followed by Scheffé’s post-hoc analysis. MCL: mitral cell layer.

NR1 deletion in DKO mice (Fig. 1) almost completely rescued brain defects in the cerebral cortex (Fig. 2), hippocampus (Fig. 3), and olfactory bulb (Fig. 4) at E16.5. In E16.5 WT mice, cerebral cortex is laminated, with the following layers: marginal zone, cortical plate (CP), subplate, intermediate zone (IZ), and ventricular zone. In the DKO cerebral cortex, the CP border on the IZ was obscured. In contrast, this abnormal laminar structure was completely restored in TKO cerebral cortex (Fig. 2). Densitometry scans demonstrated apparent border between high optical density (OD) bins in pial side and adjacent low OD bins, corresponding to CP and IZ respectively, in WT and TKO cerebral cortex. In contrast, no apparent border was observed in DKO cerebral cortex. There was a significant difference in the average OD of segment 5 (star), corresponding to the CP border on the IZ, between WT and DKO or TKO and DKO (P<0.01), but not between WT and TKO.

**Figure 5:**
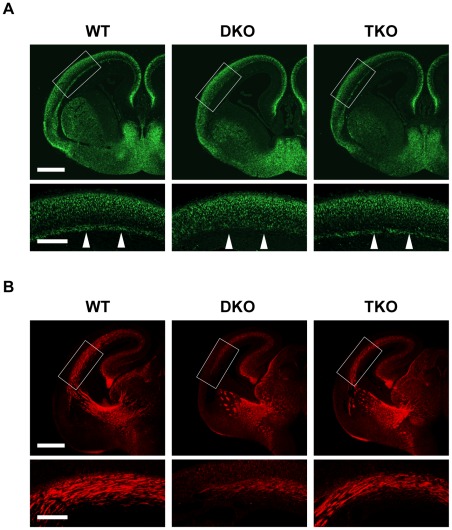
Rescue of subplate neurons and cortical connections of DKO by deletion of NR1. NR1 deletion in DKO rescued (**A**) subplate neurons (arrowheads); and (**B**) impaired cortical connections. Paraffin-embedded E16.5 coronal brain sections were stained with anti-microtubule-associated protein 2 (MAP2) (**A**), and anti-L1 antibodies (**B**). (**A**, **B**) Upper-row boxed regions are enlarged in lower row. Scale bar: upper row, 500 µm; lower row, 200 µm.

**Figure 6:**
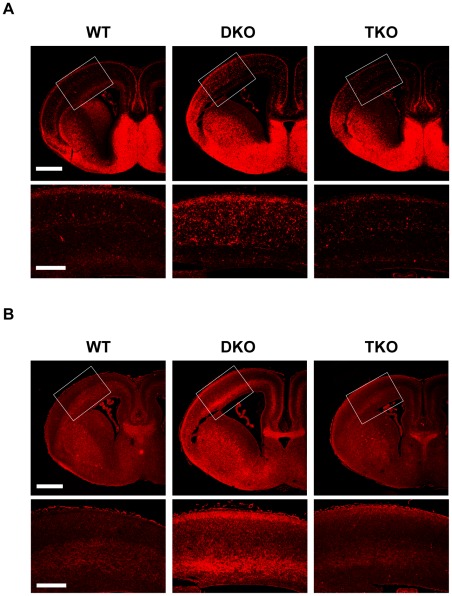
Restoration of increased levels of GAD67 and GABA in DKO by deletion of NR1. DKO showed increased levels of GAD67 and GABA. NR1 deletion in DKO reversed the increased (**A**) GAD67 and (**B**) GABA expression. Paraffin-embedded E16.5 coronal brain sections were stained with anti-glutamic acid decarboxylase 67 (GAD67) (**A**), and anti-gamma-aminobutyric acid (GABA) antibodies (**B**). (**A, B**) Upper-row boxed regions are enlarged in lower row. Scale bar: upper row, 500 µm; lower row, 200 µm.

In the DKO hippocampus, the pyramidal cell layer (PCL) was less densely packed. In contrast, abnormal PCL structure was restored in TKO hippocampus (Fig. 3). Densitometry scans demonstrated apparent OD peaks corresponding to PCL in WT and TKO hippocampus. In contrast, no OD peak was observed in DKO hippocampus. There was a significant difference in the average OD of segment 5 (star), corresponding to the PCL, between WT and DKO (P<0.01) or TKO and DKO (P<0.05), but not between WT and TKO.

In the DKO olfactory bulb, the mitral cell layer (MCL) was absent. In contrast, MCL was restored in TKO olfactory bulb (Fig. 4). The quantification demonstrated apparent OD peaks corresponding to MCL in WT and TKO olfactory bulbs. In contrast, no OD peak was observed in DKO olfactory bulb. There was a significant difference in the average OD of segment 7 (star), corresponding to the MCL, between WT and DKO (P<0.01) or TKO and DKO (P<0.05), but not between WT and TKO. These results suggest brain defects in the cerebral cortex, hippocampus, and olfactory bulb in DKO mice were almost completely rescued by NR1 deletion. Since disturbed laminar and layer organization in DKO mutants resulted from abnormal radial neuronal migration [15], these results suggested that excess NMDAR activity impaired radial migration.

Furthermore, NR1 inactivation rescued subplate neurons, which are vulnerable to early hypoxic-ischemic brain injury and important for the development of thalamus–neocortex connections (Fig. 5A). Consistent with subplate neuron rescue, severe disruptions to L1-positive corticothalamic and thalamocortical axonal projections were almost completely restored (Fig. 5B).

Recent report suggested that NR1 deletion from GABAergic neurons contributes cortical hyperexcitability and schizophrenia-associated behaviors [2]. We examined the development of GABAergic neurons using anti-GABA and anti-GAD67 antibodies. In DKO mice, the expression levels of GAD67 and GABA were increased, whereas their expression pattern was normal. In TKO mice, the elevated GAD67 and GABA expression returned to normal (Fig. 6). These results suggest that overstimulation of NMDAR can accelerate the maturation of GABAergic neurons.

Taken together, these results suggest that excess glutamatergic signaling via NMDARs, not AMPA, kainite, or metabotropic glutamate receptors, compromises early brain developmental processes.

## Discussion

Although NMDAR hypofunction does not impair embryonic brain development, its excessive function appears to be detrimental to the developing brain. Excessive NMDAR activation recapitulated schizophrenia-related pathologies in embryonic mouse brain, including enlarged lateral ventricles, disorganization of neocortex and hippocampus, and defective corticothalamic and thalamocortical axonal projections. These data raise the possibility that molecular abnormalities leading to hyper-NMDAR function in the embryonic brain could be risk factors for schizophrenia.

In contrast to glutamatergic neural circuit, excessive NMDAR activation in embryonic brain does not impair GABAergic neural circuit development. Instead, overstimulation of NMDAR can accelerate the maturation of GABAergic neurons. These results agree with the previous finding that chronic NMDA exposure accelerate development of GABAergic inhibition in the superior colliculus [21] and may be consistent with the previous result that genetic NR1 deletion in GABAergic neurons impairs the maturation of GABAergic neurons [2]. Since GABA is excitatory in the embryonic brain [22], the functional consequence of GABA and GAD67 elevations warrants future research.

Fetal hypoxia is a common risk factor for a range of neurological and psychiatric disorders including schizophrenia, autism, and epilepsy [23]. These disorders are associated with disruption of the laminar organization of the cerebral cortex. It is suggested that at least 50% of known susceptibility genes for schizophrenia are more likely than randomly-selected genes to be regulated by hypoxia [24]. Furthermore, GLT1, an “ischemia–hypoxia response gene,” is downregulated in the amygdala of patients with schizophrenia [25]. Since energy failure, as in hypoxic episodes, impairs energy-dependent glutamate transport allowing extracellular glutamate to reach excitotoxic levels [14], our results suggest that fetal hypoxia may induce neurodevelopmental abnormalities via overstimulation of NMDARs. Recently, chromosomal microdeletions of GLAST and GLT1 were linked to schizophrenia [26]–[28] and Wilms tumor, Aniridia, Genitourinary malformations and mental Retardation (WAGR) syndrome [29], respectively, and GLT1 (on 11p12-p13) was located near an autism risk locus [30]. DKO mice reproduce important pathophysiological events documented in human mental disorders, including impaired neuronal migration and cortical connections. Thus, DKO mice provide a useful tool for elucidating how embryonic disturbance of glutamate may be associated with the neurodevelopmental defects underlying neuropsychiatric developmental disorders.

Complex behavioral phenotypes such as those observed in schizophrenia and autism are hypothesized to arise from elevation in the ratio of cortical cellular excitation to inhibition (cellular E/I balance) [10]. This hypothesis could unify diverse genetic and environmental factors under a common pathophysiological principle. Our results indicate that elevated cellular E/I balance in the embryonic brain impairs brain development via hyperfunction of NMDARs. Elevation of cellular E/I balance in the adult brain by treatment with NMDAR antagonists such as ketamine and PCP mimics the symptoms of schizophrenia via the hypofunction of NMDARs in GABAergic interneurons [2], [31]–[33]. Thus, the elevated cellular E/I balance hypothesis could account for disturbance of neurodevelopment and neurotransmission in neuropsychiatric developmental disorders.

Our results suggest that offset of excessive NMDAR activity may prevent abnormal brain development due to excess glutamatergic signaling, thereby avoiding later mental disorders.

## Materials and Methods

### Animals

GLAST [34], GLT1 [35], and the NMDAR-1 subunit (NR1) [20] mutant mice were described previously. Triple-knockout mice were generated by crossing GLT1(+/−) mice with NR1(+/−) mice to obtain GLT1/NR1 double-heterozygous mice [GLT1(+/−)/NR1(+/−)], and then breeding GLAST(−/−) male mice with GLT1(+/−)/NR1(+/−) female mice to obtain GLAST/GLT1/NR1 triple-heterozygous mice [GLAST(+/−)/GLT1(+/−)/NR1(+/−)]. Finally, GLAST(+/−)/GLT1(+/−)/NR1(+/−) mice were interbred to obtain GLAST/GLT1/NR1 triple-knockout mice. All mice were of C57BL/6J background. Mice were genotyped with tail genomic DNA by described protocols for GLAST [34] and GLT1 [35]. For NR1, a set of NR1 primers for wild-type allele [36] and a set of primers for mutant allele (NR1, 5′-AGGAGTGGAACGGAATGATG-3′; Neo, 5′-CAGAAAGCGAAGGAGCAAAG-3′) were used. The day of vaginal plug detection was designated E0.5. All research and animal care procedures were approved by the Tokyo Medical and Dental University Animal Care and Use Committee (0120292C).

### Histology and Immunohistochemistry

As previously described [15], at E16.5, pregnant mice were killed by cervical dislocation, and embryos were fixed with Bouin’s fixative. Three independent embryos for each genotype were used. Paraffin-embedded sections (4 µm) were prepared. Hematoxylin staining was performed following standard protocols. Images were acquired under the constant exposure condition using a BIOREVO BZ-9000 microscope (Keyence, Osaka, Japan). For immunohistochemistry, deparaffinized sections were placed in an antigen retrieval solution (Immunosaver, Nisshin EM, Tokyo, Japan) for 45 min at 95°C and incubated with primary antibodies for 12 h at 4°C. The following antibodies were used: monoclonal anti-microtubule-associated protein 2 (MAP2) (SMI, Lutherville, MD), polyclonal anti-L1 (a gift from H. Kamiguchi, RIKEN Brain Science Institute), monoclonal anti- glutamic acid decarboxylase 67 (GAD67) (Millipore, Temecula, CA) and polyclonal anti-gamma-aminobutyric acid (GABA) (Sigma, St. Louis, MO). MAP2 was visualized by Alexa Fluor 488-conjugated goat anti-mouse IgG (Molecular Probes, Eugene, OR). L1, GAD67 and GABA were visualized by Envision-Plus Rabbit HRP System (DakoCytomation, Carpinteria, CA) and TSA plus Cyanine 3 system (Perkin-Elmer Life Sciences, Boston, MA) according to manufacturer’s instructions. Images were acquired on an LSM 510 META laser-scanning confocal microscope (Carl Zeiss, Thornwood, NY).

### Normalized Optical Density Measurement of Hematoxylin Stained Sections

To quantify the optical density (OD) of the hematoxylin stained sections, boxed regions (100 µm width) shown in Figs. 2, 3, 4 were selected, converted to 8-bit encoded grayscale and inverted to white on black, and then pixel intensity profiles along pia-ventricle axis in each region were measured using ImageJ software (3–5 sections for each embryo). In order to adjust intersectional variability of the absolute level of intensity, minimal OD value within each region was subtracted as background from individual OD values, then, individual OD values were normalized to the maximal OD value on each region. In order to adjust variations of section size, the pia-ventricular extent of the cerebral cortex, hippocampus and olfactory bulb were normalized by dividing those into 100 bins equally spaced along the pia-ventricular axis. Average values of normalized OD in each bin were plotted along the pia-ventricular axis. Three independent embryos for each genotype were used. Data are mean ± s.e.m. Statistical significance was calculated every 10 bins (segment) by one-way ANOVA followed by Scheffé’s post-hoc analysis.

### RT-PCR

Total RNA was extracted from E16.5 brain using TRIzol Reagent (Invitrogen, Carlsbad, CA). Complementary DNA (cDNA) was synthesized using PrimeScript RT reagent kit with genomic DNA Eraser (Takara, Shiga, Japan). Reverse transcription polymerase chain reaction (RT-PCR) was performed using the following primer sets: GLAST (forward, 5′-AAGCCATCATGCGATTGG-3′; reverse, 5′-CTTGAAAGTGATGGGTAGGG-3′), GLT1 (forward, 5′-CTGGTGCAAGCCTGTTTCC-3′; reverse, 5′- GCCTGTTCACCCATCTTCC-3′), NR1 (forward, 5′-GCCGATTTAAGGTGAACAGC-3′; reverse, 5′-AATTGTGCTTCTCCATGTGC-3′) and GAPDH (forward, 5′-ACTTTGTCAAGCTCATTTCC-3′; reverse, 5′-TGCAGCGAACTTTATTGATG-3′). The thermal cycler conditions were 5 min at 94°C and then 35 cycles of 20 s at 94°C, 30 s at 60°C, and 45 s at 72°C, followed by 7 min at 72°C and 60 min at 4°C.
